# VEGF signalling enhances lesion burden in KRIT1 deficient mice

**DOI:** 10.1111/jcmm.14773

**Published:** 2019-11-20

**Authors:** Peter V. DiStefano, Angela J. Glading

**Affiliations:** ^1^ Department of Pharmacology and Physiology University of Rochester Rochester New York

**Keywords:** angiogenesis, cerebral cavernous malformation, haemorrhagic stroke, vascular endothelial growth factor

## Abstract

The exact molecular mechanisms underlying CCM pathogenesis remain a complicated and controversial topic. Our previous work illustrated an important VEGF signalling loop in KRIT1 depleted endothelial cells. As VEGF is a major mediator of many vascular pathologies, we asked whether the increased VEGF signalling downstream of KRIT1 depletion was involved in CCM formation. Using an inducible KRIT1 endothelial‐specific knockout mouse that models CCM, we show that VEGFR2 activation plays a role in CCM pathogenesis in mice. Inhibition of VEGFR2 using a specific inhibitor, SU5416, significantly decreased the number of lesions formed and slightly lowered the average lesion size. Notably, VEGFR2 inhibition also decreased the appearance of lesion haemorrhage as denoted by the presence of free iron in adjacent tissues. The presence of free iron correlated with increased microvessel permeability in both skeletal muscle and brain, which was completely reversed by SU5416 treatment. Finally, we show that VEGFR2 activation is a common downstream consequence of KRIT1, CCM2 and CCM3 loss of function, though the mechanism by which VEGFR2 activation occurs likely varies. Thus, our study clearly shows that VEGFR2 activation downstream of KRIT1 depletion enhances the severity of CCM formation in mice, and suggests that targeting VEGF signalling may be a potential future therapy for CCM.

## 1 INTRODUCTION

Cerebral cavernous malformations (CCM) are characterized by thin‐walled, enlarged, multi‐chambered and leaky vascular lesions of the central nervous system. Found at an estimated rate of 0.5% in the general population, CCM‐bearing patients develop symptoms ranging from headaches, numbness and seizures, to haemorrhagic stroke. CCM development has been linked to independent loss of function mutations in 3 genes: *KRIT1* (*CCM1*), *CCM2* and *CCM3* (*PDCD10*).^1^, ^2^ However, despite the identification of several molecular targets affected by the loss of CCM proteins, the pathogenesis of CCM remains unclear. Furthermore, differences in phenotype in patients^3^, ^4^ and models^5^ with loss of function for these three genes also raise questions about common underlying mechanisms.

Blood vessel development in the mouse brain starts at E7 with the development of the earliest blood islands and terminates postnatally around P19.^6^ Thus, angiogenesis is still occurring in the brain vasculature during the first week of postnatal life, correlating with the reported developmental window for *Ccm2* deletion required to form CCM lesions.^7^ Angiogenesis is driven by the expression and activity of vascular endothelial growth factor (VEGF), a pro‐mitotic and pro‐migratory growth factor intimately linked with endothelial activation and angiogenesis. Notably, human CCM lesions have been shown to exhibit increased expression of VEGF, supporting the idea that VEGF may play a role in the pathogenesis of this disease,^8^, ^9^ as has been suggested for other types of vascular malformations, including hereditary haemorrhagic telangiectasia.^10^, ^11^ Furthermore, the abnormal morphology and increased permeability of CCM lesions is highly similar to that seen when VEGF is overexpressed in vivo.^12^, ^13^ Our previous work using an endothelial cell‐based tissue culture model showed that loss of *Krit1* and *Ccm3* stimulated the expression of VEGF. The increase in VEGF expression led to activation of VEGFR2 and subsequent increased endothelial monolayer leak, stress fibre formation, migration and phosphorylation of VE‐cadherin and β‐catenin—events required for angiogenesis and which could contribute to CCM lesion formation. Inhibition of VEGFR2 was able to acutely reverse increased microvessel permeability in *Krit1* ^+/−^ mice,^14^ supporting the idea that the VEGF pathway is up‐regulated in intact *Krit1* deficient vessels in vivo.

We hypothesized that VEGF signalling could contribute to the initiation and progression of CCM in vivo. In this study, we examined whether VEGF signalling contributes to CCM development. Using an inducible KRIT1 endothelial‐specific knockout mouse that models CCM, we show that VEGFR2 activation plays a role in CCM pathogenesis, as inhibition of VEGFR2 kinase activity reduced the number of lesions formed. More remarkable, VEGFR2 inhibition completely blocked microvessel permeability and significantly decreased the appearance of haemorrhage from lesions. Finally, we show that loss of both CCM2 and CCM3 also increases tyrosine phosphorylation of VEGFR2, suggesting that VEGFR2 activation may be a common mechanism that enhances the severity of CCM formation.

## 2 MATERIALS AND METHODS

### 2.1 Mouse models

Mice were bred and maintained under standard conditions in the University of Rochester animal facilities, which are accredited by the American Association for Accreditation of Laboratory Animal Care. All protocols were approved by the institutional review board.

Mice with a conditional (floxed) allele for *Krit1*, a germline deleted *Krit1* allele and the endothelial inducible Cre recombinase (*PDGFB‐iCreER^T2^*) have been described.^5^, ^15^ Cre recombinase activity was induced by administration of a single injection of tamoxifen (40 mg) at P1. Induced endothelial knockout mice (*Krit1^ieKO^*) contain both the floxed CCM gene allele and the endothelial inducible Cre recombinase, with no wild‐type CCM gene allele. This model is effective with either the homozygous floxed CCM gene (*Krit1^flox/flox^;PDGFB‐iCreER^T2^*, used here) or as previously described^5^ with a floxed allele combined with a null allele. *PDGFB‐iCreER^T2^; Krit1^flox/flox^* mice (*Krit1^ieKO^*), along with tamoxifen‐treated Cre‐negative *Krit1^flox/flox^* and *PDGFB‐iCreER^T2^; Krit1^flox/+^* littermate controls, were injected ip with 3 mg/kg SU5416 dissolved in corn oil (TOCRIS/R&D), or vehicle (corn oil), twice weekly, starting at P14. Treatment continued until the mice were four months of age, at which time the animals were sacrificed and perfused via the right ventricle with PBS, followed by perfusion of 3.7% formaldehyde to fix the tissue. Alternatively, male animals were prepared for intravital microscopy as described, then sacrificed and perfused.

### 2.2 Microvascular brain endothelial cell isolation

Microvascular endothelial cells were isolated from the brains of SU5416 and vehicle‐treated *Krit1^ieKO^* and control mice. Briefly, brain tissues were homogenized and then digested with 1 mg/mL collagenase A (Roche), 25 U/mL DNase (Roche) and 50 μg/mL gentamycin at 37°C for 1.5 hours, with mixing every 15 minutes. Non‐myelinated cells were pelleted through 20% BSA at 1000 *g* for 20 minutes. The cell pellet was then digested with 1 mg/mL collagenase‐dispase and 35 U/mL DNase for 1.5 hours with mixing every 15 minutes. The final cell pellet was resuspended in DMEM containing 10% FBS and layered onto a 33% Percoll (Invitrogen) gradient in 10% BSA. The gradient was separated by spinning at 400 *g* for 30 minutes, and the cell containing fraction transferred to a new tube, washed with DMEM and plated on 100 μg/mL collagen type IV‐coated plates.

### 2.3 Intravital microscopy

Preparation and visualization of the cremaster muscle, as well as measurement of permeability to Alexa‐488 bovine serum albumin (BSA, Invitrogen), was performed as previously described.^14^, ^16^ The vascular wall permeability (P_s_, cm/s) is calculated from the measured flux per unit microvessel surface area at a known BSA concentration gradient with corrections for the source volume and surface area due to the confocal slice.^17^ Images were acquired from at least 15 vessel sites, which is the appropriate determinant for n in these experiments. Data are given as combined microvessel permeability (arteriole and venule). Data were analysed using one‐way ANOVA with Bonferroni post hoc testing.

### 2.4 Fluorescein permeability assay

Ten milligrams sodium fluorescein in 0.1 mL sterile saline was injected i.p. 45 minutes prior to analysis. Serum and brain tissue were collected and assayed for fluorescein fluorescence, following trichloroacetic acid extraction and subsequent neutralization, using a BioTek Synergy H4 plate reader. Samples were compared to a standard curve of sodium fluorescein.

### 2.5 Histology

Two‐millimetre serial coronal sections of formaldehyde fixed brains were prepared and imaged using Mantis Elite HD dissecting scope (Vision Engineering) to assess total lesion number and size. Total lesion number was measured from images of the anterior plane of each 2 mm section by an investigator blind to sample identity. Lesion size was directly calculated from the image using NIH Image. The third and seventh section of each brain (anterior‐posterior) was then further processed for paraffin embedding and thin sectioning. Three 5‐μm serial sections were taken at 100 μm steps from the embedded coronal slices and stained with haematoxylin/eosin (general histology) and potassium ferrocyanide/nuclear fast red (free iron) as described previously.^5^, ^18^ Images were captured at room temperature using Infinity Capture software (Lumenera) with an UPLSAPO 10× (n.a. 0.30) objective on an Olympus IX70 microscope, and an Infinity 2 camera (Lumenera).

### 2.6 Cell culture and transfection

Human pulmonary artery endothelial cells (HPAEC, Invitrogen) were cultured in 1:1 Dulbecco's modified Eagle's medium (DMEM): F/12, supplemented with 5% foetal bovine serum (FBS), 1% endothelial cell growth supplement (ECGS, ScienCell), 1% antimycotic/antibiotic solution (Gibco/Invitrogen) and 50 μmol/L heparin (Calbiochem), at 37°C with 5% CO_2_. HPAEC were grown on 2 μg/cm^2^ gelatin‐coated tissue culture plates, and only passages 3‐6 were used in experiments. HPAEC were transfected with 30 ng siRNA using the HiPerfect transfection reagent (Qiagen) following the manufacturer's instructions. Knockdown efficiency was measured by RT‐PCR. Non‐targeting negative control siRNA #1 and anti‐*KRIT1* siRNA were obtained from Invitrogen (AM16708, Ambion/Invitrogen); *CCM2* and *CCM3* siRNAs were obtained from Dharmacon.

### 2.7 VEGF enzyme‐linked immunoassay (ELISA)

ELISA high binding plates (Thermo Fisher Scientific) were coated with 1 μg/mL of mouse anti‐VEGF (R&D Biosystems) in phosphate‐buffered saline (PBS) overnight at room temperature. Plates were then blocked with 300 μL of blocking buffer (1% BSA in PBS) for 1 hour at room temperature. After washing with wash buffer (PBS with 0.2% Tween‐20), 100 μL samples of conditioned medium from siRNA‐transfected 60 mm plates were added to appropriate wells and incubated at room temperature for 2 hours. A standard curve of recombinant VEGF from 0 to 2000 pg/mL was run alongside experimental samples. Plates were washed again and incubated with 1.2 μg/mL goat anti‐VEGF (R&D) for 2 hours at room temperature. After washing, streptavidin‐horseradish peroxidase (HRP, Thermo Fisher Scientific) was added for 1 hour followed by detection with 3,3′,5,5′‐tetramethylbenzidine (TMB, eBioscience). Data were analysed using one‐way ANOVA with Tukey's post hoc testing.

### 2.8 Immunoprecipitation and western blotting

HPAEC or mouse brain microvascular cell lysates were prepared in MgALB150 lysis buffer (50 mmol/L Tris pH 7.4, 150 mmol/L NaCl, 0.5% NP‐40, 5 mmol/L MgCl_2_, phosphatase and protease inhibitors). Protein quantification was performed using a bicinchoninic acid assay kit (Thermo Fisher), and equivalent amounts of total cell protein were added to tubes containing 2 μg monoclonal rabbit anti‐VEGFR2 and 20 μL of protein G‐Sepharose beads. After incubation at 4°C overnight, immunoprecipitated proteins were blotted against phospho‐tyrosine (Cell Signaling) and then re‐probed with anti‐VEGFR2 to assess precipitation efficiency. Alternatively, lysates were directly probed with rabbit anti‐phosphoY1175 VEGFR2 (Thermo Fisher Scientific), followed by incubation of secondary antibody (goat anti‐rabbit DyLight‐800 (Fisher)). An Odyssey Infrared Imaging System (LI‐COR Biosciences) was used to image membranes and for densitometry.

### 2.9 Statistics

Statistical analysis (ie one‐way ANOVA with appropriate post hoc testing) was performed using PRISM software (version 4.0, GraphPad Software Inc). Statistical significance was assessed assuming a .05 significance level and a two‐sided alternative hypothesis.

## 3 RESULTS

### 3.1 VEGFR2 inhibition reduces lesion number in Krit1 endothelial knockout mice

**Figure 1:**
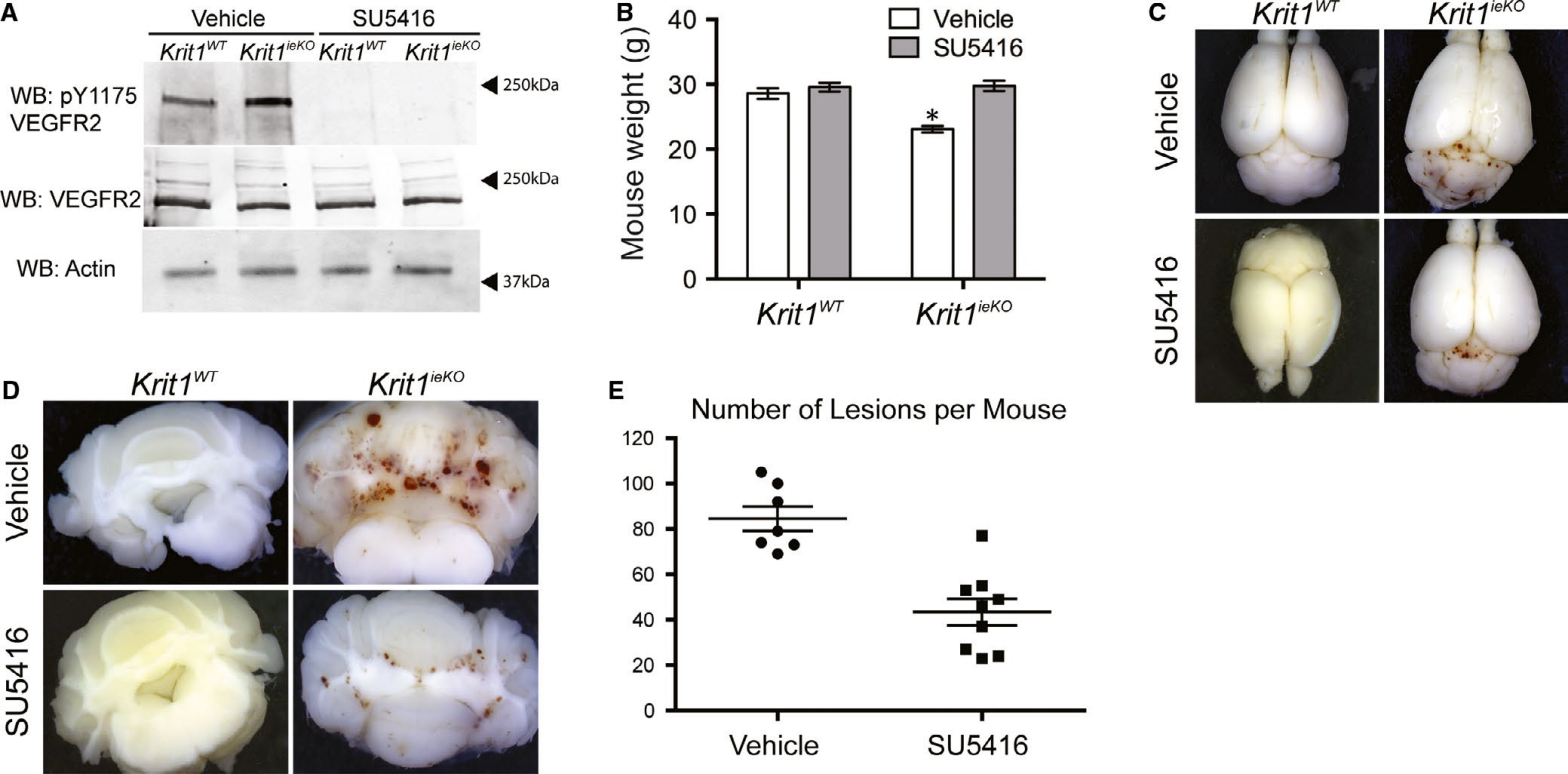
VEGFR2 Inhibition Reduces Lesion Number in *Krit1^ieKO^* mice. A, Tyrosine 1175 phosphorylation (pY1175), total VEGFR2 and actin expression in microvascular brain endothelial cells isolated from wild‐type (*Krit1^WT^*) or *Krit1^ieKO^* mice treated with vehicle or SU5416. Blots are representative, n = 3. B, Average mouse weight (g) at 16 wk of age ± SEM, *P = .008 vs vehicle‐treated *Krit1^ieKO^* mice by 2‐way ANOVA. C, Representative gross images of whole brains from wild‐type or *Krit1^ieKO^* mice. Lesions are noticeable at the junction between the cerebrum and cerebellum. 4× magnification. D, Representative images of 2 mm coronal slices of brains from *Krit1^WT^* or *Krit1^ieKO^* mice. 4× magnification. E, Average number of lesions per mouse from vehicle or SU5416 treated *Krit1^ieKO^* mice. Data shown are mean ± SEM, n = 7, *P* = .0013 by unpaired *t* test

Our in vitro data strongly support the idea that VEGF is a critical player in the development of CCM. To test this directly, we utilized *Krit1^ieKO^* mice, which develop lesions similar to those seen in human CCM patients.^15^ Tamoxifen injection of *PDGFB‐iCreER^T2^Krit1^flox/flox^* mice at P1 stimulates endothelial‐specific Cre‐mediated recombination and loss of Krit1 protein in >90% of the endothelium. In these animals, Type 1 lesions (enlarged vessels with a single lumen but no apparent haemorrhage) are visible to the naked eye in >90% of animals at 2 weeks of age (Figure S1). Mature multi‐cavern lesions are found in 100% of animals at 3 months, and death occurs around 5 months of age (data not shown). To test whether VEGF signalling downstream of loss of *Krit1* is involved in CCM pathogenesis, we treated *Krit1^ieKO^* mice with 3 mg/kg of the VEGFR2 inhibitor SU5416 twice weekly until 4 months of age, starting at P14. SU5416 (Semaxinib) is a potent and effective VEGFR2 kinase inhibitor and has been shown to inhibit angiogenesis in several in vivo models.^19^, ^20^, ^21^ *KRIT1^ieKO^* mice exhibited increased pY1175 VEGFR2 levels in cerebral endothelial cells compared to their wild‐type counterparts, which was reversed following SU5416 treatment (Figure 1A), demonstrating the effectiveness of the inhibitor in vivo. Animals treated with SU5416 exhibited higher weights (Figure 1B) and developed significantly fewer CCM‐like lesions (Figure 1C‐E, Vehicle: 84.57 ± 5.41 vs SU5416: 43.44 ± 5.88) which was apparent upon gross inspection.

### 3.2 VEGFR2 inhibition does not limit lesion size

**Figure 2:**
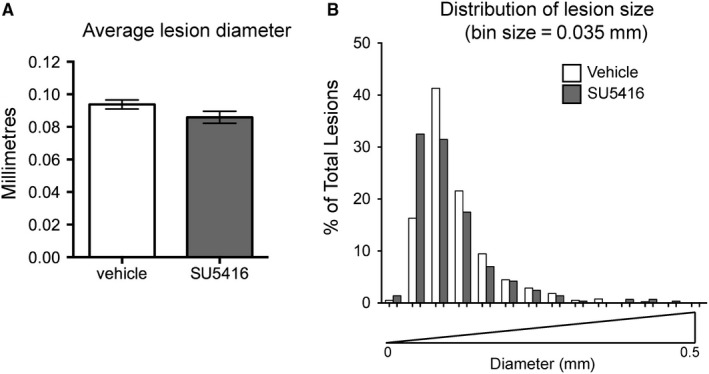
Distribution of lesion size in vehicle and SU5416 treated *Krit1^ieKO^* mice. A, Average maximum lesion diameter ± SEM. Vehicle, n = 593; SU5416, n = 391. B, Histogram distribution of percent total lesion number by lesion diameter (mm)

Previous studies have linked lesion size to the progression of CCM, as smaller lesions in the human population appear less likely to become haemorrhagic/symptomatic.^2^ Indeed, *Krit1^ieKO^* mice as well as *Ccm2* and *Ccm3*‐dependent mouse models of CCM show a range of lesion size and complexity.^5^ There was no statistical difference in average lesion size in SU5416‐treated *Krit1^ieKO^* mice compared to vehicle control (Figure 2A), and the distribution of lesion sizes was roughly equivalent (Figure 2B). Thus, while inhibition of VEGFR2 decreases the number of lesions, it does not significantly affect the distribution of lesion size, suggesting that VEGF may be more important for the initiation, but not the growth, of CCM lesions.

### 3.3 VEGFR2 inhibition blocks vascular permeability in Krit1^ieKO^ mice

**Figure 3:**
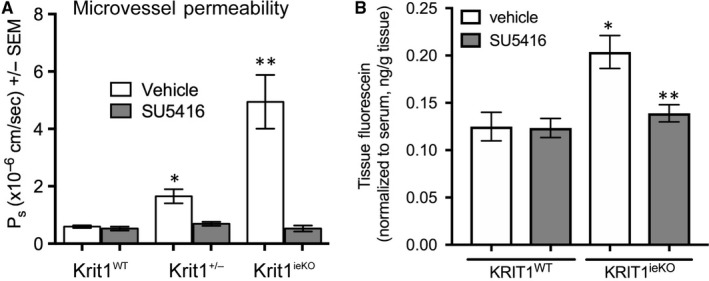
VEGFR2 activation increases in vivo microvessel permeability following loss of KRIT1. A, Cremaster microvessel permeability in wild type (*Krit1^WT^*), heterozygous (*Krit1* ^+/−^) or *Krit1^ieKO^* treated with vehicle or SU5416. Data shown are mean P_s_ ± SEM, n = 15 vessel sites, *P* < .001 by ANOVA, *P < .001 by post hoc testing vs vehicle treated *Krit1^WT^* and **P < .001 vs vehicle‐treated *Krit1^ieKO^*. B, Permeability of brain vasculature to fluorescein. Data shown are mean tissue fluorescence, normalized to serum fluorescein levels ± SEM, n = 4 mice/group, *P* < .01 by ANOVA, *P < .01 by post hoc testing vs vehicle treated *Krit1^WT^* and **P < .001 vs vehicle‐treated *Krit1^ieKO^*

One of the most debilitating aspects of CCM is haemorrhagic stroke caused by chronic or acute blood leakage from lesions.^2^ Our previous work indicated that inhibition of VEGF signalling in *KRIT1* hemizygous cells and animals could acutely restore permeability to control levels.^14^ We hypothesized that the loss of both alleles of *Krit1* in *Krit1^ieKO^* animals would have a more profound impact on vascular permeability, which could be reversed by inhibition of VEGF signalling. Therefore, we examined cremaster muscle microvessel permeability in *Krit1^ieKO^* animals treated with vehicle or SU5416 for 14 weeks, starting at P14. As expected, vehicle‐treated *Krit1^ieKO^* animals exhibited over a fourfold increase in microvessel permeability compared to *Krit1*‐expressing control animals. This increase was completely inhibited by treatment with SU5416, and indeed, the permeability of SU5416 treated *Krit1^ieKO^* animals was reduced below control levels (Figure 3A). As CCM lesions predominate in the central nervous system, though clearly the effect on vessel integrity extends beyond the brain in our mouse model, we also investigated whether VEGF inhibition affected the permeability of fluorescein in the brains of KRIT1 deficient mice. *Krit1^ieKO^* animals exhibited a 63% increase in the permeability of fluorescein versus control animals, which was reversed by treatment with SU5416 (Figure 3B).

### 3.4 VEGFR2 inhibition reduces lesion haemorrhage

**Figure 4:**
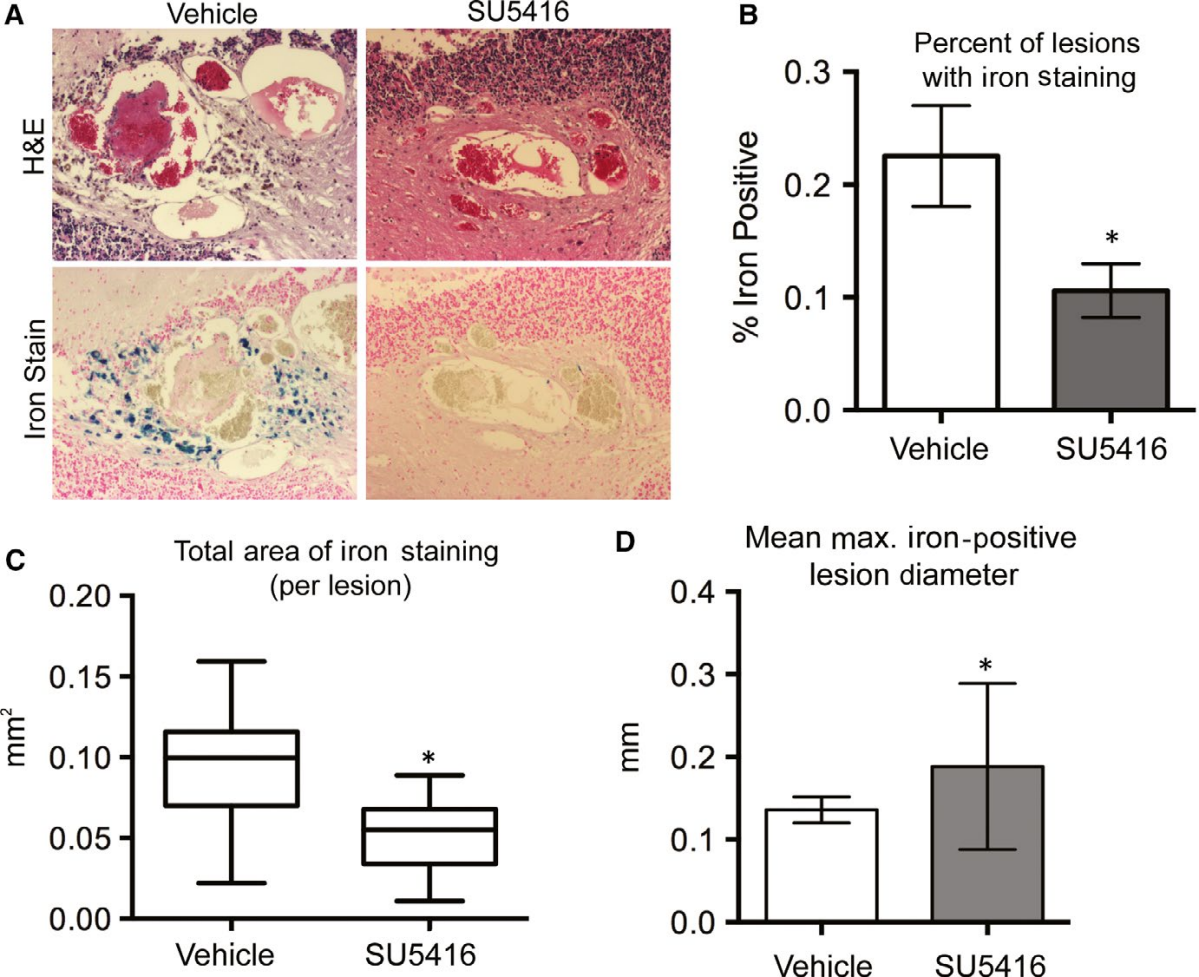
VEGFR2 activation increases lesion haemorrhage following loss of KRIT1. A, Haematoxylin and eosin (H&E) and free iron staining of 5‐µm sections of coronal brain slices from *Krit1^ieKO^* mice treated with vehicle or SU5416. Images are representative. Iron staining appears blue in image. B, Percentage of lesions that are positive for iron staining from vehicle and SU5416 treated *Krit1^ieKO^* mice. Data shown are averaged from 2 distinct 5‐µm sections per mouse ± SEM, n = 11 per treatment type, *P = .017 by unpaired *t* test. C, Average area of iron staining per lesion. Box is standard deviation, with mean ± 95% CI (whiskers). Vehicle, n = 117; SU5416, n = 32. *P = .002 by unpaired *t* test. D, Average maximum lesion diameter of iron‐positive lesions. Vehicle, n = 117; SU5416, n = 32. *P = .03 vs vehicle treated by unpaired *t* test

As increased permeability may contribute to the rate of haemorrhage in CCM lesions, we next wanted to assess the appearance of haemorrhage in the lesions of vehicle and SU5416 treated animals. Haemorrhage in human and mouse CCM lesions can be observed by staining for free iron, a product of extravascular red blood cell lysis. We observed a significant decrease in the appearance of free iron in the lesions of SU5416 treated Krit1^ieKO^ animals (0.11% ± 0.02) when compared to vehicle‐treated Krit1^ieKO^ (0.24% ± 0.04, Figure 4A,B). In addition, the extent of iron staining in vehicle‐treated animals greatly exceeded that of SU5416 treated animals, suggesting that the total extent of haemorrhage was severely limited by the inhibition of VEGFR2 (Figure 4C). As larger lesions are thought to be more likely to haemorrhage, we assessed whether the reduction in iron‐positive lesions was due to a reduction in the number of larger lesions by examining the size distribution of lesions that were iron positive. In vehicle‐treated *Krit1^ieKO^* mice, iron‐positive lesions had an average diameter of 0.14 ± 0.02 mm. However, in SU5416 treated *Krit1^ieKO^*, the majority of iron‐positive lesions were larger (0.19 ± 0.02). This is clearly shown as a shift in the median size of iron‐positive lesions (Figure 4D), suggesting that inhibition of VEGF/VEGFR2 activation decreases the likelihood of haemorrhage of small to medium lesions, but not large lesions, a potentially clinically significant finding.

### 3.5 Loss of all CCM protein family members increases VEGFR2 tyrosine phosphorylation

**Figure 5:**
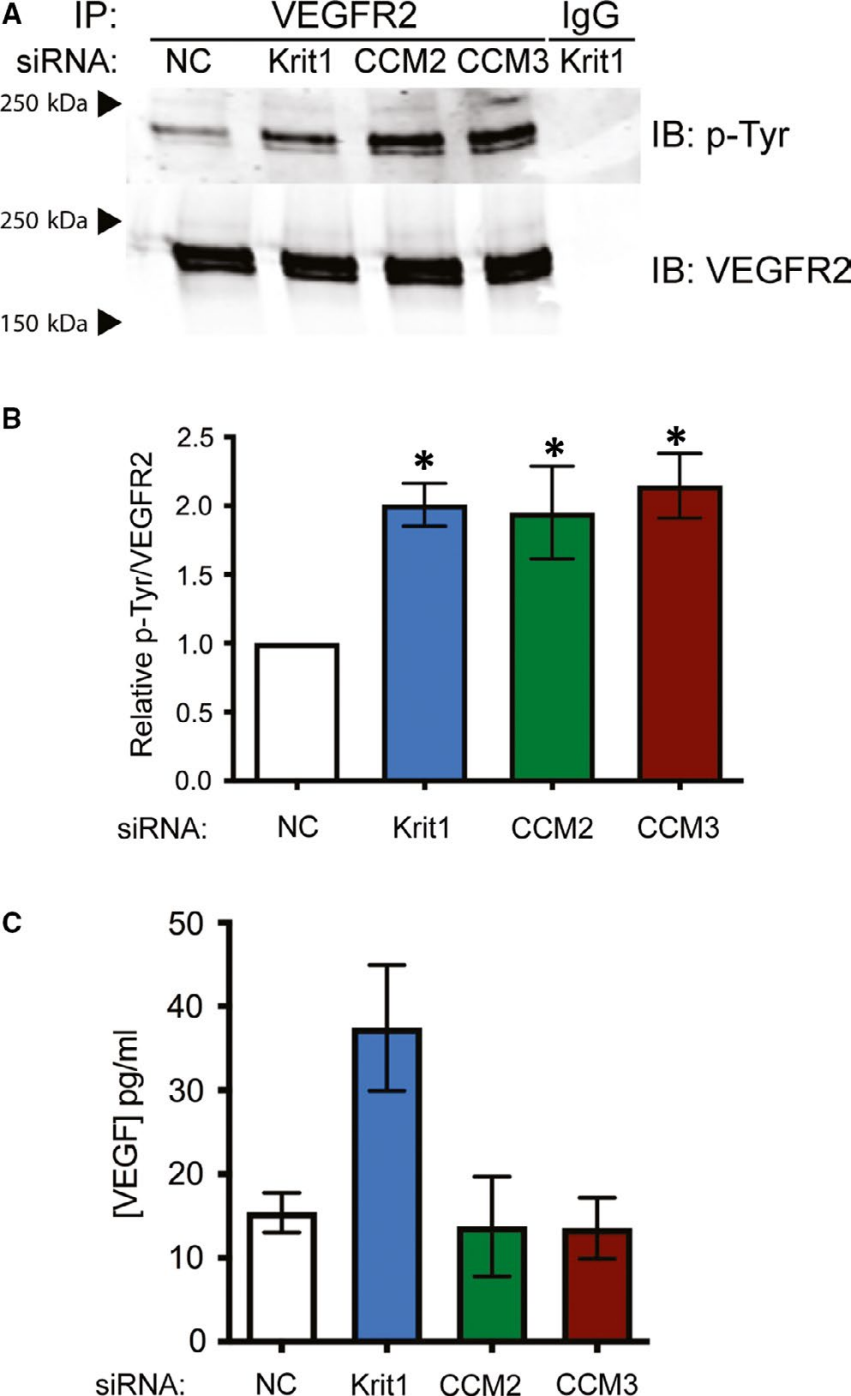
Loss of CCM protein family members increases VEGFR2 tyrosine phosphorylation. A, Tyrosine phosphorylation of VEGFR2 in HPAEC transfected with negative control (NC), anti‐*KRIT1*, anti‐*CCM2* and anti‐*CCM3* siRNA. IB, immunoblot; IP, immunoprecipitation. Blots are representative, n = 3. B, Densitometric quantification of blots in (A). Data shown are P‐Tyr normalized to total VEGFR2, ± SEM, *P* = .0223 by ANOVA, *P < .05 by post hoc testing vs NC transfected cells. C, VEGF protein (pg/mL) in conditioned media harvested from cells expressing anti‐*KRIT1*, anti‐*CCM2* and anti‐*CCM3* siRNA *P* = .032 by ANOVA, *P < .05 by post hoc testing vs NC transfected cells

An association between the genetic loss of CCM proteins and VEGF signalling has previously been suggested, though the mechanism underlying this connection remained unclear. We recently found that loss of *KRIT1* and *CCM3 *in vitro induces an increase in *VEGF* mRNA via increased ß‐catenin/TCF‐dependent nuclear transcription. In *KRIT1* depleted cells, the subsequent increase in VEGF protein and activation of VEGFR2 contributed to an activated endothelial phenotype with several hallmarks of angiogenic endothelium.^14^ As the up‐regulation of *VEGF* mRNA led to increased VEGFR2 activation in *KRIT1* depleted cells, we predicted a similar effect in *CCM3* but not *CCM2* depleted endothelial cells, which did not up‐regulate *VEGF* mRNA.^14^ Interestingly, independent knockdown of both *CCM3* and *CCM2* elevated tyrosine phosphorylation of VEGFR2 in human pulmonary artery endothelial cells (Figure 5A,B and Figure S2), but did not increase VEGF secretion (Figure 5C). These data suggest that VEGFR2 activation may be a common signalling mechanism in CCM development, though the mechanisms are likely divergent.

## 4 DISCUSSION

Blocking endogenous VEGFR2 activation significantly reduced the number of lesions that formed within the brain of *Krit1* deficient animals (Figure 1). Our previous study demonstrated the presence of a feed‐forward mechanism whereby loss of *KRIT1* caused increased nuclear β‐catenin transcriptional activity, increased expression of VEGF and activation of VEGFR2 to promote loss of barrier function, migration, etc. We have now shown that the activation of VEGFR2 is a common mechanism downstream of depletion of all three CCM proteins (Figure 5A). This was surprising in light of our previous finding that *CCM2* knockdown showed no significant increase in *VEGFA* mRNA, and though *CCM3* knockdown did increase *VEGFA* mRNA,^14^ it did not increase VEGF protein secretion (Figure 5C). However, the clear presence of activated VEFGR2 in *CCM2* and *CCM3* deficient cells suggests that an alternative mechanism exists to promote receptor activation downstream of loss of these proteins. One such mechanism was reported by You et al who showed that down‐regulation of Dll4/Notch signalling in *CCM3* depleted endothelial cells increased VEGF and VEGFR2 expression.^22^ However, another study found that VEGFR2 activity was diminished in the absence of *CCM3*.^23^ These discrepancies may be due to experimental differences (ie knockdown level, activation/inactivation of specific tyrosines on VEGFR2) and remain to be reconciled. Alternatively, recent reports have demonstrated a role for inflammatory signalling and oxidative stress in CCM lesion development, including the up‐regulation of the oxidative stress‐responsive transcription factor Nrf2,^24^ which indirectly promotes VEGF expression.^25^ Furthermore, Tang et al noted that activation of Toll‐like receptor‐4 (TLR4) signalling by blood‐borne gram‐negative bacteria led to greater CCM lesion burden in a mouse model of CCM.^26^ In addition to activation of TLR4, infection with gram‐negative bacteria can be associated with increased expression of VEGF.^27^, ^28^ Our data are consistent with these observations. Though more work needs to be done to elaborate the mechanism underlying VEGFR2 activation in the absence of CCM proteins, our work supports the potential impact of VEGF signalling induced by infection as an important disease modifier and opportunity for therapeutic intervention.

Our data strongly suggest that activation of VEGF signalling could be a common element of CCM pathogenesis. This suggests that angiogenic environmental signals, perhaps combined with cell autonomous changes in gene expression, fundamentally alter the phenotype of the endothelium to cause lesion genesis. However, activation of VEGF signalling could also contribute to the variation in the clinical presentation of CCM, or the progression of the disease. Local differences in the activation of VEGF signalling could provide the necessary context for lesion development. However, this fails to specifically address the stochastic development of lesions, which could be due to changes in other genetic modifiers or local sensitivity to vascular stress.^29^, ^30^, ^31^, ^32^ Strikingly, inhibition of VEGFR2 completely blocked the increase in endothelial permeability caused by loss of *Krit1*, which translated into a significant decrease in haemorrhage from lesions. These data support the idea, as suggested by clinical studies, that VEGF could be a prognostic indicator of haemorrhage risk in CCM patients. On the other hand, VEGF inhibition had no effect on the distribution of lesion size, indicating that this mechanism likely does not affect the growth or expansion of CCM lesions. To be effective, a potential treatment should affect at least one of the three key features of CCM disease: lesion initiation, lesion growth or lesion haemorrhage. Our data strongly suggest that inhibition of VEGFR2 can address lesion formation and haemorrhage, 2 of the 3 cardinal features.

The inability to fully block lesion formation in vivo may alternatively reflect the need to manipulate several pathways simultaneously, though which pathways to select remains an elusive answer. Intriguingly, several of the pathways modified following loss of *KRIT1* (or *CCM2*) are known to crosstalk with VEGF signalling. For example, loss of both *KRIT1* or *CCM2* increases phosphorylation of ERK1/2,^33^, ^34^ which is also activated downstream of VEGFR2.^35^ Also, loss of *KRIT1* or *CCM2* leads to an increase in active c‐Jun N‐terminal kinase (JNK).^31^ The activation of JNK in *KRIT1* depleted cells is mediated by reactive oxygen species, which can be activated by VEGF signalling,^36^ or activate VEGF signalling.^37^ More studies to outline the hierarchy of these pathways will be necessary before we can determine whether all should be targeted, or whether, for example, VEGF signalling could be targeted as a common mediator, as is suggested by our data.

These observations have important implications for the therapeutic approach to CCM disease. Currently, the mainstay of therapy for CCM is surgical excision or radiological destruction of the lesion. There are now several potential therapeutic approaches suggested to impact CCM lesion pathophysiology, and it is hoped that a new era of medical management of CCM will prove beneficial. Anti‐angiogenic therapy may prove useful to prevent or delay lesion formation, or to stabilize the haemorrhage prone lesion and may form part of a multi‐pronged medical approach to the treatment of CCM.

## CONFLICT OF INTEREST

The authors have no conflicts to declare.

## AUTHOR CONTRIBUTIONS

AJG conceptualized and supervised the project, and wrote the manuscript. PVD and AJG performed experiments. PVD analysed data and prepared figures. All authors contributed intellectually and reviewed the manuscript.

## Data Availability

The data that support the findings of this study are available from the corresponding author upon reasonable request.
