# Supplementary figures and images for: VEGF signalling enhances lesion burden in KRIT1 deficient mice
Source: J Cell Mol Med. 2019 Nov 20;24(1):632–9. doi: 10.1111/jcmm.14773 (PMC6933401; doi:10.1111/jcmm.14773)

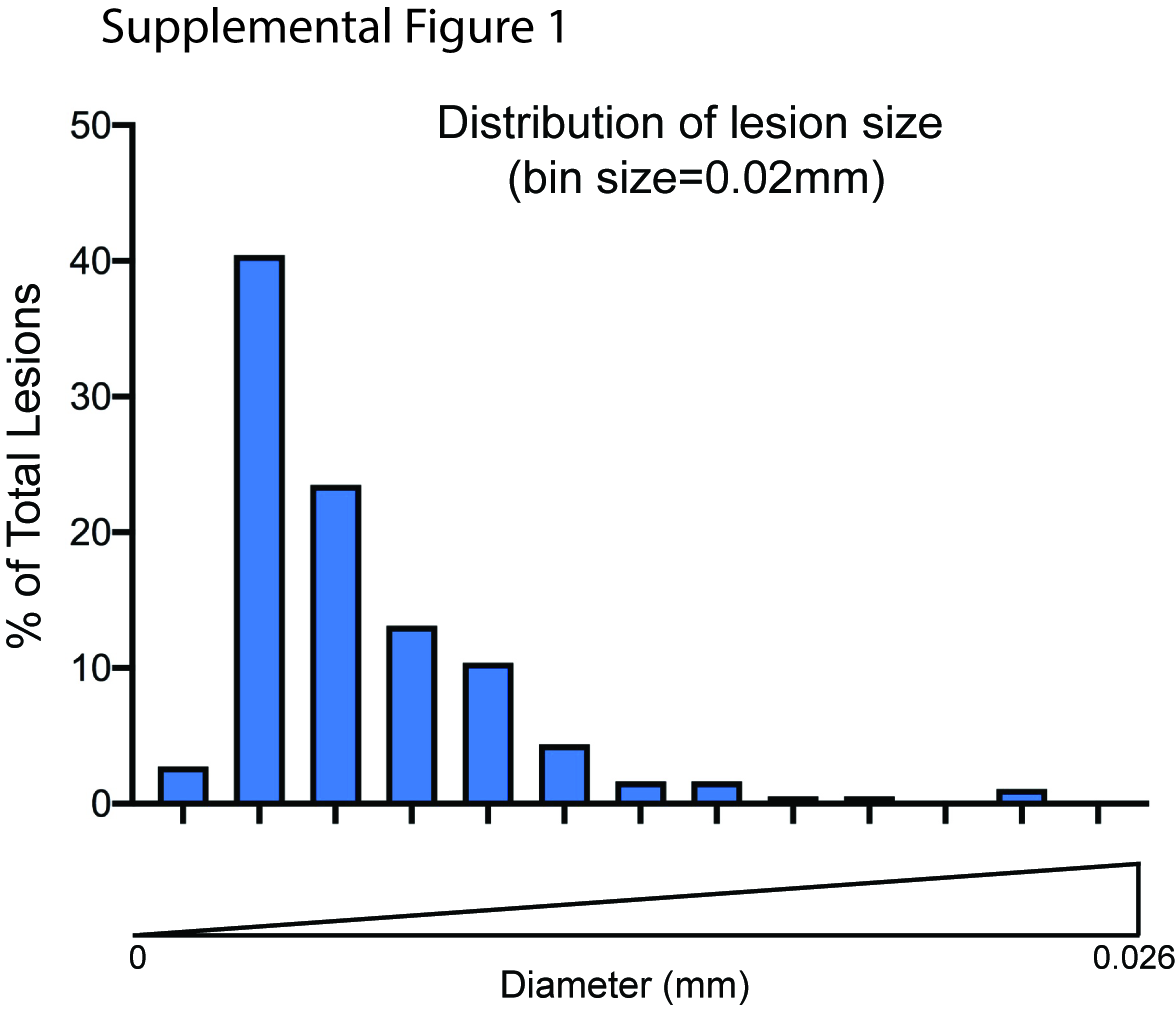

Supplement: Supplementary file 1 [file JCMM-24-632-s001.tif]

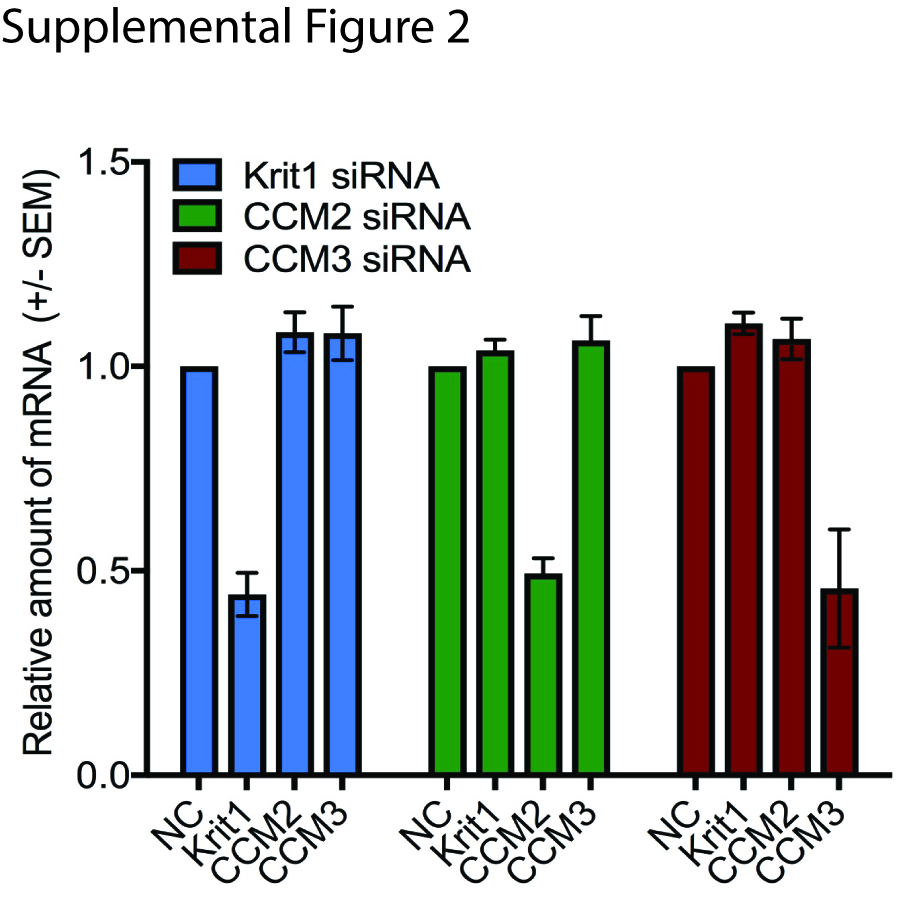

Supplement: Supplementary file 2 [file JCMM-24-632-s002.tif]
